# 

**Published:** 1805-12

**Authors:** 


### INDEX.

ABDOMEN, case of fungous tumour on the 79

Abernethy's, Mr. lectures announced 285

Academicus, on the case of the child of 1

Achromatic glasses recommended 12

Acids to be used in purifying infected atmosphere 205

—— in scarlatina, on 373

Ackworth, scarlatina in the school at 374

Adams's, Dr. Tract on Cow-pox, account of 96, 377

—————— on the cancerous breast 82

—————— on vaccination 193

—————— case of small-pox 353

—————— Mr. Ring's answer to 355

Addenbrooke's Hospital, Cambridge, account of 344

Addington's, Mr. misstatements on vaccination 433

Affusion, on cold, in fever 203

—————— in influenza 226

Agues, the water-flag a remedy for 67

———— on solutio arsenici in 100

Air, apparatus for purifying the 190

—— on the influence of the 570

Albumen, characteristics of 263

Alchemy, description of a manuscript on 91

Ale made by the Kamschatkadales 171

—— brewed in the Highlands 542

Alkalies, their effects on poisons 478

————, in fever, on 373

Allen's Mr. lectures announced 285

America, account of travels in 187

Amicus on a diseased testicle 529

Amputation at the shoulder-joint, case of 108

——————, on the article of, in the Cyclopædia 111

Aphthous ulcers in the mouth, cure for 546

Anatomical injection, description of a new 52

Anderson, Mr. on tumours of the brain 81

Aneurism, on the article in the Cyclopædia, on 111

——————, on the practice of the Greeks in 112

Anger, epilepsy following a fit of 275

Animal fluids, analysis of the 263

Antiscorbutic, a good 65

Apartments, on lighting 13

Apoplexy, on bleeding in 379

Apparatus for purifying the air, described 190

Aqua frigida, its use in scalds, &c. 239

Argand's lamps recommended 14

Aristolochia, its benefit in chlorosis 170

Armies, on the medical department of 549

Armiger's, Mr. demonstrations announced 383

Arnemann, Dr. undertakes the foreign department of the Medical and Physical Journal 192

—————— his observations and cases 430

Arsenic, in hydrophobia, remarks, on 30

————, in agues, efficacy of 100

————, in eruptions 113

Arteries, on the 52

Articulations, on unnatural 475

Asparagus, botanical description of 66

Asphodel, description of *ib.*

Asthma, a good expectorant for 65

Atmosphere, on the pressure of the 213

——————, on purifying infected 205

——————, of London, pollution of 184

Aubert, Dr. on vaccine inoculation 401

Augurapory, account of an alchemic treatise so called 91

Babington's, Dr. lectures announced 285

Bacon, Lord, on empiricism 440

Badham, Dr. on the pneumonic diseases of the poor 83

——————, lectures announced 286

Baillie, Dr. on tumours of the brain 81

Baldinger, Prof. account of his medical library 192

Barberry, botanical description of the 69

Barclay, Dr. on a peculiar structure of the urethra 469

Bark, its efficacy in rheumatism 273

——, its efficacy in yellow fever 531

—— for tanning leather, account of a new 572

Barlow's, Mr. case of tinea capitis 496

Bartholomew's Hospital, lectures at 285

Bateman's, Dr. case of tumour of the brain 80

Bathing in fever, on 87, 179, 343

————, its efficacy in agues 101

Batty's, Dr. lectures announced 286

Becu, Dr. on vaccination 149

Bell's second volume of surgery announced 283

Bennion, Mr. on the Gibraltar fever 136

Berger's, Dr. case of necrosis of the tibia 300

Berlin, prize questions of the medical society at 381

Bilberry bush, botanical description of 173

Binoculum recommended for preservation of the eyes 12
Binns's, Dr. obfervations on vifion 152
Birch, Mr. on failures of the cow-pox, reviewed 376
———, a correction of a miftake of 480
Birthwort, climbing, botanical defcription of 170
Bifhopp's, Mr. cafe of optical illufion 117
——— rheumatifm 118
——— remarks on 328
Bizet, Mr. on the fap in plants 209
Bladder, remedy for the ftone in the 546
Blair's, Mr. cafe of fmall-pox after vaccination 21
———, author of articles in the Cyclopædia 111
———, lectures announced 286
Bleeding, its fuccefs in hydrophobia 122
———, its inefficacy in the influenza 227
Blizard's, Sir W. lectures announced 383
Blood, on the difcovery of the circulation of the 74
———, on the vital principle of the 51
Blood-letting, its danger in apoplexy 379
Blue girl, cafe of a 471
Books, critical analysis of new medical. Coup d'Œil fur les Revolutions et fur la Reforme de la Medicine, par P. J. G. Cabanis, 73. Edinburgh Medical and Surgical Journal, 78, 263, 469. Addrefs to the Medical Practitioners of Ireland, by S. B. Labatt, M. D. 84. Addrefs delivered at the Small-pox and Inoculation Hofpitals, previous to the Funeral of Wm. Woodville, M. D. by Anthony Highmore, 85. Dr. Jackfon on the Treatment of Fevers, 85, 179. Obfervations on late Attempts to depreciate the Value and Efficacy of Vaccine Inoculation, by Samuel Merriman, 174. Cafes of two extraordinary Polypi removed from the Nofe, by Thomas Whately, 179. Salutary Cautions refpecting the Gout, by John Hunt, 260. A Clinical Hiftory of Difeafes, Part I. by John Haygarth, M. D. 272. Defcription and Treatment of Cutaneous Difeafes, Order III. by R. Willan, M. D. 362. An Epitome of Infantile Difeafes, by J. Smyth, M. B. 374. A Letter occafioned by the many Failures of Cow-pox, by John Birch, 376. Anfwers to all the Objections made againft Cow-pox, by Jofeph Adams, M. D. 377. Cafes of Pulmonary Confumption treated with Uva Urfi, by Robert Burne, M. D. 461. Enquiry into the Nature and Action of Cancer, by Samuel Young, 465. Syftem of Arrangement and Difcipline for the Medical Department of Armies, by Robert Jackfon, M. D. 549. Obfervations on the fimple Dyfentery, by William Harty, M. B. 557. Cow-pox Inoculation no fecurity againft Small-pox, by William Rowley, M. D. 565. The Modern Practice of Phyfic, by Edward Goodman Clarke, M. D. 569.
Books, new medical announced. Dr. Trotter on Nervous, Bilious, &c. Difeafes, 95. Dr. Adams on Morbid Difeafes and Cow-pox, 96. Dr. Jones on fuppreffing Hæmorrhage from divided and punctured Arteries, *ib.* Dr. Jackfon's Enquiry into the Nature of the Gibraltar Fever, *ib.* Dr. Harty's Obfervations on the Dyfentery, *ib.* Dr. Garnett's Zoonomia, *ib.* Dr. Kinglake's Cafes of Gout, 192. Mr. Saunders on the Structure of the Ear, 283. Tranfactions of the London Medical Society, *ib.* Mr. Donovan's Natural Hiftory of the Infects of New Holland, &c. *ib.* The fecond volume of Bell's Surgery, *ib.* Index to the firft Series of Medical and Phyfical Journal, 288. Mr. Ring's Anfwer to Dr. Mofeley, 384. Mr. Luxmore's Manual of Anatomy, *ib.* Dr. Fauft and Dr. Hunold on Surgical Inftruments, 480.
Borda, Dr. on the laurocerafus 572
Boftock's, Dr. analyfis of animal fluids 263
Botanical defcription of Britifh plants 62, 169, 541
Bougies, on the advantages of cauftic 474
Bourne, Dr. on uva urfi in pulmonary confumption 461
Bradley's, Dr. lectures announced 286
Brain, cafe of encyfted tumour on the 80
———, cafe of difeafed 247
———, cafe of collapfed 289
———, on the bony covering of the 298
———, Dr. Gall's new doctrine of the 327
———, cafes of contufion of the 486
Breaft, cafe of tumour of the 8[illegible]
Breflaw, inoculation at 235
Briggs's, Mr. treatife on the difeafes of the eyes announced 283
Brookes's, Mr. lectures announced 287
Broth, preferved for years by mercury 479
Brown's, Dr. lectures announced 283
———, Dr. D. letter on the yellow fever 389
———, on the effects of cold on the human body 241
Bruffels, prize queftions of the Medical Society at 382
Bryony, botanical defcription of 68
Buchholz's, Dr. preparation of naptha aceti 383
Burton's, Dr. cafe of hydrophobia 122
Cabanis, M. fur les Revolutions et fur la Reforme de la Medicine, reviewed 73
Cancer in both tefticles, cafe of 83
———, review of Mr. Young's treatife on 465
Candles, on the placing of 13
Cantharides, their inefficacy in tetanus 12[illegible]

Carmichael's, Mr. cafe of ftricture in the urethra 174
Carpue's, Mr. lectures announced 287
Catheter, on the introduction of the 449
——, defcription of a canulated 490
Catfkill, in America, account of the yellow fever at 313
Cauftics, on the application of 105
Celfus, on fecuring blood-veffels 103
Centegrade, enquiry concerning the 576
Ceylon, vaccination in the ifland of 409
Chamberlaine's, Mr. communication on the Yellow Fever 185
Charcoal, method of making 546
Chatard's, Dr. cafe of ftrangulated hernia 206
Chevalier's, Mr. lectures announced 287
Chicken-pox, cafe of confluent 141
Child, on the cafe of a 1
Chirurgicus, anfwers to 40, 103
Chlorofis, benefit of ariftolochia in 170
Chreftien, Dr. on inoculation 142
Chronic fyphilitic complaint, cafes of 516
Cinchona, in rheumatifm, on 326
Clark, Dr. of Newcaftle, character of 285 *note*
Clarke's, Dr. lectures announced 287
Cleghorn's, Dr. lectures announced 283
Cline's, Mr. lectures announced 285
Cold, cafe of torpor from 267
——, advantages of, in ophthalmia 184
—— bathing in fever, on 87
——, effects of, on the human body 239
Coleman's, Mr. lectures announced 285
Collins's, Mr. cafe of tympanites 267
Conium maculatum, on the ufe of 125
Confumption, on uva urfi in pulmonary 461
Contagion, on 386
——, on deftroying 341, 573
Contufion of the brain, cafes of 486
Convulfions, on puerperal *ib*
Cooke's, Dr. lectures announced 383
Cooper's, Mr. cafe of fungous tumour in the abdomen 78
—— lectures announced 285
—— letter to Mr. Cribb 246
Copal varnifh, true method of making 382
Corns, how to deftroy 64
Correfpondents, to 96, 192, 288, 384, 480
Cofmetic, an excellent 67
Cotton, Dr. anecdote of 370, *note*
Cow inoculated with vaccine virus 29
Cow-pock, Dr. Winterbottom on the inoculation of 22
——, on the blue 27
——, queries on the 95
——, Dr Sims on the 480
Cow-pox, addrefs to the inhabitants of Norwich on 277
—— inoculation no fecurity againft fmall-pox, reviewed 565
——, letter on the failures of, reviewed 376
Cow-pox, anfwers to all the objections made againft 377
——, fee Inoculation and Vaccination.
Coxe's, Dr. cafe of tetanus 129
Cranberries, defcription of 173
Crane, Dr. on a cafe of impregnation 416
Cribb's, Mr. fingular cafe of delivery 17
——, anfwer to Mr. Dawfon 242
Cullen, Dr. on the effects of cold on the human body 241
Cuming, Mr. Cuftance's anfwer to 1
——, reply of Mr. Wilkinfon to 46
——, on fcrofula, &c. 36
——, on the ufe of aqua frigida 239
——, on the management of leeches 448
——, on ifchuria 449
Cuprum ammoniacum, preparation of 47
Curry's, Dr. lectures announced 285
Cuftance, Mr. on the death of a child 1
Cutaneous difeafes, account of Dr. Willan's treatife on 362
——, garlic applied in cafes of 65
Cynanche, inefficacy of bleeding in 184
Dalrymple's, Mr. cafe of trifmus 267
Dancer, Dr. on the yellow fever 385
Darlington, extraordinary fpring near 191
Davis's, Dr Tranflation of Penil's Treatife on Infanity, announced 573
Dawfon's, Mr. obfervations on a cafe of delivery 155
—— on recent depreffions of the skull 161
—— reply to, by Mr. Cooper 242
Deafnefs, efficacy of garlic in 65
De Carro, Dr. extract of a letter from 283
Delivery, fingular cafe of 17
——, obfervations on 155
Denman, Dr. on cutting the frænum of the tongue 97
Dennifon's, Dr. lectures announced 96, 286, 383
Dentifrice, an excellent 71
Desks, on placing writing 12
Diabetes mellitus, morbid appearances in two cafes of 269
Diemerbroeck, on a recurrence of fmall-pox 195
Digitalis, its efficacy in hydrocephalus 238
Dimnefs of fight, cafe of 15
Dimfdale, Baron, on inoculation 311
Difeafes in the Finfbury Difpenfary, from May 20 to June 20 14
—— from June 20 to July 20 183
from July 20 to Auguft 20 275
—— from Aug. 20 to Sept. 20 378
—— from Sept. 20 to Oct. 20 476
—— from Oct. 20 to Nov. 20 570
——, on mental 276

Difeafes, review of Haygarth's hiftory of clinical 272
—— of the rich and poor, on the 276
Dobeimer's, Mr. method of making white lead 383
Dock, botanical defcription of the different kinds of 70
Dog, on the Colthorpe remedy for the bite of a mad 31
——, Sir T. Mayerne's recipe for the bite of a mad 32
Donovan's, Mr. natural hiftory of infects, announced 283
Drefs, advice with refpect to 380
Dropfy, a diuretic for the 65
——, cure for the 170
——, obfervations on 571
Dudley, on vaccination at 433
Duhamel, M. on the fap in plants 209
Duncan's, Dr. analyfis of bone 471
Dunning, Mr. on inoculation 53, 308
Dupuytren's Mr. account of an extraordinary fœtus 157
Dwight, Dr. on the yellow fever 312
Dyfentery, remedy for the 70
——, obfervations on fimple 557
Dyfpepfia, cafe of 477
Ear, treatife on, announced 283
Earneft's, Mr. cafe of fingultus 514
—— fungus ulcer *ib.*
—— chronic fyphilitic complaint 516
Earth, on the preffure of the atmofphere on the 213
Edinburgh, medical and furgical journal, reviewed 78, 263, 469
Education, on the improvements of medical 75
Edwards's, Dr. lectures announced 285
Effluvia of the human body, on the 387
Egremont, letter from the earl of 574
Electricity, its efficacy in gutta ferena 481
Empiric, account of an antient 442
Empiricifm, on the origin and progrefs of 437
Epidemical fever, obfervations on 337
—— ophthalmia, on the effects of cold in 240
Epilepfy, efficacy of fugar of lead in 10
——, following a paroxyfm of anger 275
——, on Galvanifm in 527
Errata 192, 288, 384, 576
Eruption, effects of arfenic in 113
Exercife, on intellectual and bodily 277
Experiments on animal fluids 264
—— on muriatic acid 272
Eye, cafe of a bony tumour in the 471
Eyes, on the management and prefervation of the 11, 152
Face, cafe of eruption of the 116
Fafhions in philofophy 387, *note*
Fauft, Dr. on furgical inftruments 480
Favus, on the eruption called 198
Felix, Dr. on ol. tereb. in burns 398
Ferriar, Dr. on hyfteria 395
Fever, Dr. Jackson, on the treatment of 85, 179
——, at Gibraltar, on the 136, 200, 202
——, on epidemical 337
——, on cold affufion in 203, 226
——, report of the inftitution for preventing 340
——, on mercurial inunction in 38
——, on fcarlet 368
Finfbury difpenfary, report of difeafes in the 14, 183, 275, 378, 476, 570
——, election of a furgeon for the 384
Flatulent colic with hernia, cafe of 271
Flax, botanical defcription of 62
Fluids, analyfis of animal 263
Forbes, Dr. on empiricifm 437
Fothergill, Dr. on the ulcerous fore throat 369
Foundling hofpital, vaccination and re-inoculation at the 506
—— hofpitals, on the utility of 271
Fox's, Mr. lectures announced 285
Fracture cured by nitric acid 29
Frænum of the tongue, on cutting the 97, 256
Frampton's, Mr. lectures announced 286
Freckles, how to remove 64
Freer's, Dr. lectures announced 283
Friefe's, Dr. letter on vaccination 233
Fungus ulcer, cafe of 514
Funis umbilicalis, cafe of impervious 483
Galen, on binding the veins and arteries 106
——, on empirics 440
Gall's, Dr. doctrine of the brain and mental faculties 327
Galvanifm, its effect in epilepfy 527
Garlic, botanical defcription of 64
Garnett, Dr. on hydrocephalus 45
—— Zoonomia, notice of 96
Gaffner's, Dr. cafe of the bite of a fnake 524
Gaubius on deftroying contagion 573
George's, St. hofpital, lectures at 286
Germany, ftate of vaccination in 26
Geftation, on 180
Gibbes, Dr. anecdote of 408
Gibraltar fever, on the 136, 200
Gillefpie's, Mr. communication on the krameria triandria 127
—— letter on the ftate of health in Lord Nelfon's fleet 412
Glafgow, medical lectures in the univerfity of 283
——, regulations of the fenate of 284
Goetling's improved preparation of kermes mineral 112
Goodall's, Mr. cafe of hernia 452
Gout, fuccefsful application of cold water in 120
——, benefit of the ariftolochia in 171

Gout, Dr. Kinglake's anfwer to Mr. Mantal on 215
———— treatife on, announced 192
——, Mr. Mantal in reply, on 455, 497
——, falutary cautions refpecting the 260
Gravel, remedy for the 65
Grew, Dr. on the fap in vegetables 209
Griffiths, Mr. on inoculation 5
Gum, refemblance between mucus and 266
Gums, remedy for putrid 71
Gunning's, Mr. lectures announced 383
Gutta ferena, cafe of 481
Guy's hofpital, lectures at 285
Hæmorrhage cured by the krameria triandria 128
Haighton's, Dr. lectures announced 285
Hall, Mr. on fuffocation 150
———— on vaccination 410
———— his attempt to improve the fcrew tourniquet 492
Hamilton's, Dr. new faline draught 53
———— lectures announced 383
Harrold, Mr. on a cafe of rheumatifm 323
Harrifon's, Mr. propofal of a Jennerian circle 230
Harty's, Dr. obfervations on the dyfentery announced 96
Harty, Dr. on fimple dyfentery, reviewed 557
Hawker, Mr. on epidemical fever 337
Haygarth's, Dr. clinical hiftory of difeafes reviewed 272
Hazel-nut tree, defcription and ufes of 545
Headington's, Mr. lectures announced 286, 383
Health, eftablifhment of a board of 191
Heart, on the ftructure of the 298
——, cafe of organic affection of the 517
Heath, common, defcription and ufes of 541
Heberden, Dr. on variolous inoculation 7
———— on electricity in gutta ferena 482
Hemlock, on the leffer 428
Hendy's, Dr. cafe of hyfteric phrenitis 391
Hepatitis, cafe of 469
Hernia, on a cafe of ftrangulated 206
——, cafe of, treated with opiates 271
——, Mr. Goodall's cafe of 452
——, cafe of incarcerated 454
Herophilus, the father of the empirics 444
Higgins's, Dr. centegrade and hygrometer, enquiry concerning 576
Highmore's, Mr. addrefs at the interment of Dr. Woodville, reviewed 85
Hill, Mr. on inoculation 48
Hippocrates's aphorifms, editions of 192
Holftein, defcription of cow-pock in 27
Holway, Dr. on the cow-pox *ib.*
Home's, Mr. lectures announced 383
Honey from heath, defcription of 542
Hooper's, Dr. lectures announced 383
Hooping cough, remedy for 39
Hornbrook, Mr. on ol. tereb. in burns 34
Huggan, Dr. on mercury in hydrophobia 538
Human body, effects of cold on the 241
Humboldt and Bonpland's travels, account of 187
Hunt's, Mr. falutary cautions on gout, reviewed 260
Hunter, Mr. on the incompatibility of two actions 262
Hyacinth, botanical defcription of the 67
Hydrargyrus vitriolatus, its ufe in gutta ferena 482
Hydrocephalus, fingular cafe of 42
———— internus, cafe of 237
Hydrogen fufceptible of different degrees of oxydation 272
Hydrophobia, on arfenic in 30
———— treated by bleeding and mercury 122
————, on mercury in 538
Hygrometer, enquiry concerning 574
Hypochondriac, fingular account of a 277
Hyfteria, optical illufion from 117
Hyfteric phrenitis, cafe of 391
Idlenefs, on 276
Illufion, cafe of optical 117
Impregnation, cafe of 17
————, remarks on 416
Index to the Medical and Phyfical Journal, notice of 288
Infantile difeafes, epitome of, reviewed 374
Influenza, on cold affufion in 226
Inglis, Dr. on unnatural articulations 476
Injection, account of a new anatomical 52
Inoculation, Mr. Griffith on 5
————, Mr. Ring on *ib.* 142, 355, 401
————, Mr. Blair on 21
————, Mr. Hill on 48
————, Mr. Dunning on 54, 308
————, Mr. Magin on 57
————, Mr. Little on 58
————, Mr. Smith on 60
————, queries on 95
————, Mr. Merriman on 174
————, Dr. Adams on 193, 353, 377
————, Dr Friefe on 233
————, addrefs to the inhabitants of Norwich on 277
————, on variolous 310
————, Dr. Hall on 410
————, Mr. Wainwright on 433
Inftruments, furgical, advice refpecting 480
Intermittents, on 99
Inunction, efficacy of mercurial 38
Ireland, addrefs to the medical practitioners of 84
————, on vaccine inoculation in 437
————, on the medical police of 574
Ifchuria, on 448
Itch, cure for the 70
Jackfon, Dr. on the treatment of fevers 85, 179

Jackson, Dr. his treatise on the Gibraltar fever, announced 76

———, Dr. on the medical department of armies reviewed 549

Jardine's, Mr. case of collapsed brain 289

Jaundice, a remedy for the 69

Jelly, characteristic of 266

Jenner, Dr. letter from the Earl of Westmeath to 256

———, anecdotes of 407

Jennerian society, committee appointed by the 191

———, report of the royal Sussex 346

———, address of the royal Somerset 348

——— circle, proposal of a provincial 230

Joints, on the nodosity of the 275

Jones's, Dr. treatise on hæmorrhage, announced 96

Kamtschatkadales, ale and vinegar made by the 171

Kellie's, Dr. case of torpor from cold 267

Kelly, Dr. on the functions of the skin 84

Kentish, Dr. on the use of ol. terebinth. in burns 396

Kermes mineral, improved preparation of 112

Kinglake's, Dr. explanation of a case 72

———, additional cases announced 192

———, reply to Mr. Mantal 215

———, Mr. Mantal's answer to 455, 497

Krameria triandria, on the virtues of the 127

Labatt's, Dr. Address to the medical practitioners of Ireland, reviewed 84

Lafuente's, Dr. method of curing yellow fever 531

Lancet, on abolishing the 183, 306

Lang, Dr. on the preservation of the eyes 11

Laplanders, food of the 72

Laurel, spurge, description and properties of 545

Laurocerasus, on the effects of 572

Lawrence's, Mr. lectures announced 285

Lazarettoes recommended 205

Lead, on the efficacy of sugar of 10

LECTURES announced: Drs. Dennison and Squire on Midwifery and the Diseases of Women and Children, 96, 286. In the University of Glasgow, 283. Dietetics, Materia Medica, and Pharmacy, by Dr. Millar, *ib.* Midwifery, by Mr. Towers, *ib.* Dr. Freer on the Theory and Practice of Physic, *ib.* Dr. Jeffray on Anatomy and Surgery, *ib.* Dr. Cleghorn on Chemistry, *ib.* Dr. Freer's Clinical Lectures, *ib.* Dr. Brown's on Botany, *ib.* St. Bartholomew's Hospital, 285, viz. Dr. Roberts and Dr. Powell on the Theory and Practice of Medicine; Mr. Abernethy on Anatomy, Physiology, and Surgery; Mr. Macartney on Comparative Anatomy and Physiology; Dr. Edwards on Chemistry; Dr. Powell on the Materia Medica; Dr. Thynne on Midwifery and the Diseases of Women and Children. Mr. Lawrence's Anatomical Demonstrations, 285. St. Thomas's and Guy's Hospitals, viz. Anatomy and Surgical Operations by Mr. Cline and Mr. A. Cooper; Principles and Practice of Surgery by Mr. Cooper; Practice of Medicine by Dr. Babington and Dr. Curry; Principles and Practice of Chemistry by Dr. Babington and Mr. Allen; on Midwifery by Dr. Haighton; Pathology, Therapeutics, and Materia Medica, by Dr. Curry; Physiology by Dr. Haighton; Experimental Philosophy by Mr. Allen; Clinical Lectures by Drs. Babington, Curry, and Marcet; Veterinary by Mr. Coleman; on the Teeth by Mr. Fox, 285. St. George's Hospital, Dr. Pearson on Therapeutics and the Practice of Physic, 286. London Hospital, Mr. Headington and Mr. Frampton on Anatomy, Physiology, and Surgery, 286, 383. Mr. Armiger's Anatomical Demonstrations, *ib.* Dr. Dennison on Midwifery, *ib.* Dr. Cooke on the Theory and Practice of Physic, 383. Drs. Hamilton and Yelloly on Chemistry, *ib.* Sir William and Mr. T. Blizard's Clinical, 383. Westminster Hospital, 286. Dr. Badham's on Medicine, Chemistry, &c. *ib.* Dr. Batty's on Midwifery, *ib.* Mr. Blair's on the Natural History of Man, *ib.* Dr. Bradley's, on the Theory and Practice of Physic, *ib.* Mr. Brooke's, on Anatomy, Physiology, and Surgery, 287. Mr. Carpue's, on Anatomy, *ib.* Mr. Chevalier's, on Surgery, *ib.* Dr. Clarke's, on Midwifery, *ib.* Mr. Milburne's Physiological, *ib.* Dr. Reid's, on the Theory and Practice of Medicine, *ib.* Mr. Taunton's, on Anatomy and Surgery, *ib.* Dr. Squire's, on Midwifery, 288. Mr. Thelwall's, on Elocution, *ib.* Mr. Wilson's, on Anatomy, &c. *ib.* Mr. Home's, on Surgery, 383. Mr. Gunning's, on Syphilitic Complaints, *ib.* Dr. Hooper's, on the Theory and Practice of Physic, *ib.* Mr. Moor's, on the Teeth, 384. Dr. Reid's, on the Theory and Practice of Medicine, *ib.* Mr. Thomas's, on Surgery, *ib.*

Leeches, on the management of 186

Leefe's, Mr. account of the second appearance of small pox 251

Leg, on the necrosis of the tibia of the right 300

Lettsom's, Dr. communication respecting an alchemic MS. 91

Literary men, on the preservation of the sight of 154

Ligature, on the introduction of the, 40, 103, 111

Light, on the effects of 153

Lily of the valley, botanical description of 66

Lime, on the muriate of, in the cure of scrophula 80

Linseed, properties of 63

Little, Mr. on inoculation 58

Livingstone's, Mr. case of cancer of both testicles 83

London, account of the medical and chirurgical society of 191

——— hospital, lectures at the 283

——— medical society, volume of their transactions announced 383

——— impurity of the atmosphere of 184

Luxmore's, Mr. Manual of Anatomy announced 384

Lymphatics, on a disease of the 50

Macartney's, Mr. lectures announced 285

Magin, Mr. on inoculation 57

Mantal, Mr. reply to by Dr. Kinglake 215

——— his answer 455, 497

Marcet's, Dr. lectures announced 285

——— case of a blue girl 471

Martin, Dr. on tapping at the navel 418

Mayerne's, Dr. recipe for the bite of a mad dog 32

Measles, co-existence of small-pox and 142

———, on inoculation for the 362

Medical practitioners not to be severely treated 3

———, education on 75

——— and Chirurgical Society instituted 191

——— and physical intelligence 91, 185, 277, 381, 478, 572

——— lectures, see Lectures

Medicine, on the study of 3, 74

——— alliance between moral philosophy and 76

Medicines, new theory on the effects of 572

Mercurial inunction in fevers, on 38

Mercury, its efficacy in hydrophobia 122

Mercury in hydrophobia, on 538

Merriman's, Mr. observations on attempts to depreciate vaccination 174

Mezereon, description and uses of 544

Milburne's, Mr. lectures announced 287

——— plan of instruction 576

Millar's, Dr. lectures announced 283

Mind, its ascendancy over material organization 16

Mind, on the disorders of the 276

Mogalla, Dr. a convert to vaccination, 233

Monro's, Dr. case of hepatitis 469

Moor's, Mr. lectures announced 384

Mortality, bill of at Vienna 383

Morveau's fumigations adopted in Prussia 382

Moseley's, Dr. cases, replies to 5, 177

Mouth, remedy for ulcers in the 546

Mucus, experiments on 265

Muriatic acid, on the composition of 272

———, method of using it in purifying the air 205

Myrtle flag, botanical description of 67

Nabob, anecdote of a 380, *note*

Naphtha aceti, improved method of preparing the 383

Naval surgeons, regulations respecting 38

Navel, on the advantage of tapping at the 418

Necrosis of the tibia, case of 300

Needle, on the early use of in hæmorrhages 111

Nelson, Lord, letter to 412

Nervous diseases, treatise on announced 95

——— affections, on the 275

Nerves, on the origin of 329

Night, question on its influence on the sick 382

Nitric acid, fracture cured by the use of 29

———, method of using it for purifying the air 205

Nodosity of the joints, on 275

Norwich, memorial addressed to the inhabitants of 277

Oil, surgical instruments should be dipped in 480

——— cake, on 63

Ol. terebinth. its efficacy in burns 34

Opiates, flatulent colic with hernia cured by 271

Opium, cases of rheumatism treated with 81

Opium, its effects in the bite of a snake 524

Ophthalmia, on cold water in 184, 241

Optical illusion, case of 117

Organization, ascendancy of the mind over material 16

Orkney islands, account of the influenza in the 226

Otto, M. on arsenic in eruptions 113

Pacchioni, Prof. on muriatic acid 272

Palmer's, Mr. case of hydrocephalus internus 237

Paré, Ambrose, his claim to the invention of the ligature 103

Parker, Mr. on leeches 186

Parsley, hemlock mistaken for 427

Patterson, Dr. on vaccination 437

Patterson, Dr. on pestilential and epidemic diseases 574

Pearfon's, Dr. lectures announced 286
Penel, Dr. on nitric acid in fracture 29
Peffary, advantages of the fponge 98
Pewter not noxious 33
Pharmaceutical procefs 112
Pharmacy, on 276
Philenus one of the earlieft empirics 444
Phlegmon, rheumatifm producing 118
Phrenetis, cafe of hyfteric 391
Phyfic, caution with regard to the ftudy of 3
Phyficians, on the duties of 77
Plague, on the contagioufnefs of the 390
Plants, botanical defcription of Britifh 62, 169, 451,
——— on the motion of fap in 289
——— on poifonous 425
Plowden's, Mr. cafe of fmall-pox 404
Plymouth-dock, mortality of the fmall-pox at 310
Poifon, from toadftools, remedy for 573
Poifonous plants, obfervations on 425
Poifons, effects of alkalies in 478
Polypi, cafes of extraordinary 179
Poor, on the pheunomic difeafes of the 83
———, on the diforders of the 276
Pope's, Mr. cafe of hernia 454
Poplar tree, defcription and ufes of 542
Portland powder, ingredient in the compofition of the 171
Potafh, on the properties of 382
Powell's, Dr. lectures announced 285
Prolapfus uteri, cafe of 98
Prouft, M on the properties of potafh 382
Provincial Jennerian circle propofed 230
Prurigo, defcription of 525
Pruffia, regulation for the prevention of the yellow fever in 382
Pulley's, Mr. cafe of amputation 108
Pulley, Mr. on the bony covering of the brain 529
Pulmonary complaints, obfervations on 83
——— on the virtues of uva urfi in 461
——— inflammation, hyfteric phrenetis fupervening on 391
Purgative, an excellent 63
Queries on the cow-pox and fmall-pox 95
——— on the fting of a wafp 479
Queftions prize, of the medical fociety at Berlin 381
——— of the fociety at Bruffels 382
Ramfay, Mr. on a difeafe incident to the lymphatics 50
——— his new anatomical injection 52
Ramfon garlic, botanical defcription of 65
Rafori's, Dr. new theory on the effects of medicines 572
Rattle fnake, experiments on the poifon of the 479
Reeves's, Dr. hiftory of a tumour in the breaft 82
Reid's, Dr. report of difeafes in the Finfbury Difpenfary 14, 183, 275, 378, 476, 570
——— lectures announced 287, 384
Relatives, on the lofs of 4
Refufcitation, cafe of 7
Rheumatifm, an excellent purgative in 63
——— cafes of acute, treated with opium 81
——— producing phlegmon, cafe of 118
——— remarks on 323
——— on cinchona in 326
——— hiftory of acute 273
Rheumatifm, efficacy of fpurge laurel in 545
Rich, on the difeafes of the 276
Ring, Mr. on inoculation, 6, 112, 509
——— his cafe of confluent chicken pox 141
——— letter from Dr. Friefe to 233
——— anfwer to Dr. Adams 355, 401
——— his anfwer to Dr. Mofeley announced 384
Roberts's, Dr. lectures announced 285
Roches, Dr. on opium in rheumatifm 81
Rofs, Mr. on the Gibraltar fever 290
——— Dr. his cafe of flatulent colic 271
Rouffeau on bleeding 184
Ruiz, Don, on the plant krameria triandria 127
Rufh's, Dr. cafe of refufcitation 7
———, on fugar of lead in epilepfy 10
——— on a cafe of hydrophobia 124
Rutherford's, Dr. account of morbid appearances in diabetes mellitus 268
Saffron, meadow, defcription of 169
Saline draught, account of a new 53
Sap in plants, on the motion of 289
Saunders's, Mr. Treatife on the ear announced 283
Scalds, on cold water in 239
Scarlatina on 366
Schlegel, Dr. on viola tricolor in venereal ulcers 431
Schmidt, Dr. on the organ of the eye 11
Scrophula, on 36
———, on muriate of lime in the cure of 80
Scrofulous ophthalmia, on the application of cold in 184
Scurvy, remedy for the 71
Sea air, its advantages in the fummer 184
Sea bathing, its efficacy in agues 101
Sedum acre, its virtues in epileptic fits 430
Shearly's, Mr. cafe of gout 126
Sheep-pox, on the 142
Sheernefs, badnefs of the barracks at 100
Shillito, Mr. on intermittents 99
Shoulder joint, cafe of amputation at the 108
Sight, remarkable cafe of dimnefs of 15
———, advice on the prefervation of 154

Simmons, Mr. on the invention of the needle and ligature 103

—— on the extermination of the fmall pox 107

—— his felect cafes of furgery 481

Sims, Dr. on the fpurious cow-pock 480

Simpfon's, Mr. cafe of prolapfus uteri 98

Singultus, cafe of 514

Skin, on the functions of the 82

Skull, on depreffions of the 161

——, Dr. Gill's lectures on the 327

Small-pox, after vaccination, cafes of 5, 21, 23

—— on a fecond appearance of the 176, 251

—— on the extermination of the 107, 140, 454

—— hofpital, cafes at the 194

Smith, Mr. on inoculation 60

Smyth's, Dr. epitome of infantile difeafes reviewed 374

Snake, effects of opium in the bite of a 524

Snow, on rubbing the body with 91

Solomon's feal, botanical defcription of 67

Somerfet Jennerian Society, addrefs of the 348

Sorbus accuparia, its bark ufed in tanning 572

Sorrel, botanical defcription of 71

Sparrows, fagacity of the 382

Speech, on defects of 259

Sponge peffary, advantages of the 98

Squire's, Dr. lectures announced 96, 288

Stevens on poifonous plants 425

Stone in the bladder, remedy for 546

Stringham's, Dr. cafe of hydrocephalus 42

—— obfervations on the yellow fever 79

Struve, Dr. on an alchemical manufcript 99

Suffocation, cafe of 150

Sugar of lead, its efficacy in epilepfy 10

—— made from the fap of the fycamore 172

——, yellow fever cured by fweating in the fteam of 185

Sulphur fpring, difcovery of a 191

Summer, impurity of the London atmofphere in 184

Sun-burn, how to remove 64

Sun-dew, botanical defcription of the 64

Surgeons, regulations refpecting naval 38

Surgery, felect cafes of 481

Surgical inftruments fhould be dipped in oil 480

Suffex Jennerian Society, report of the 346

Sweating, yellow fever cured by 185

Sycamore tree, botanical defcription of 172

Sydenham, on the fmall-pox 196

Tanning, new procefs of 572

Tapping at the navel recommended 418

Taunton's, Mr. lectures announced 287

—— elected furgeon of the Finfbury difpenfary 384

Tefticle, cafe of difeafed 529

Tefticles, cafe of cancer of both 83

Tetanus, inefficacy of cantharides in 129

Thelwall, Mr. on cutting the bridle of the tongue 256

—— opens a feminary at Liverpool 288

Thermometer, its defects in medical application 86

——, defcription of a new 572

Theffalus, an empiric, account of 442

Thomas, Dr. on the Gibraltar fever 202

——, Mr. lectures announced 384

——'s St. Hofpital lectures at 285

Throat, on the ulcerous fore 369

Thynne's, Dr. lectures announced 285

Thyroid cartilage, cafe of its becoming ulcerated 516

Tibia, cafe of necrofis of the 300

Tin, quantity of arfenic in 33

Tinea capitis, fatal cafe of 305

—— its effects in vaccination 6

—— on the mode of curing the 496

Toadftools, remedy againft the effects of 573

Tobacco, its fatal effects in tinea capitis 305

Tongue, on cutting the frænum of the 97, 256

Torpor from cold, cafe of 267

Tourniquet, on the fcrew 492

Towers's, Mr. lectures announced 283

Trail, Mr. on the influenza 226

Tranfpiration in plants on 213

Trotter's, Dr. Treatife on nervous difeafes announced 95

Tumour, encyfted in the brain, cafe of 80

——, in the breaft, cafe of 82

Ulcer, cafe of fungus 514

Ulcerated fore throat, obfervations on the 369

Ulcers, mezereon applied to 544

—— effects of viola tricolor in venereal 431

—— in the mouth, remedy for 546

Urethra, peculiar ftructure of the membrane lining the 469

—— cafe of ftricture in the 474

Urinary bladder, child contained in the 519

Urine, cafe of fuppreffion of 252

Uteri, cafe of prolapfus 98

Uva urfi, its efficacy in pulmonary confumption 461

Vaccination, cafes of fmall-pox after 5, 21

——, addrefs to the practitioners of Ireland, on 84

——, queries on 95

——, Dr. Adams on 193

——, Dr. Hall on 410

——, Mr. Wainwright on 433

——, in Ireland, account of 437

——, at the Foundling Hofpital, account of 506

——, at Gottingen, account of 535

——, remarkable inftance of failure of 536

Vaccination, see INOCULATION.

Vaccine inoculation, on some late attempts to depreciate 174

———, Earl of Westmeath's letter on 256

Valentin's, Dr. memoirs of Dr. Jenner, correction of 407

Van Swieten on the ligature 41

Variolation, failure of 536

Variolous inoculation, on restraining 140

———, Dr. Heberden on 7

Varnish, method of making copal 382

Vaughan, Dr. on arsenic in hydrophobia 30

Vegetables, on the sap in 209

Venereal disease, efficacy of conium maculatum in 126

——— ulcers, effects of viola tricolor in 431

Venæsection, on the danger of 183

——— observations on 306

——— French too much addicted to 226

Vienna, deaths in ten years at 383

——— small-pox exterminated at 408

Vinegar made by the Kamschatkadales 171

———, method of making balsamic and anti-putrid 573

Viola tricolor, its effects on venererl ulcers 431

Vision, observations on 152

Wadley, Mr. on venæsection 306

Wainwright, Mr. on vaccination 433

Walker, Dr. on the bony covering of the brain 298

———, remarks on 529

Warts, how to destroy 64

Wasp, on the poison of the 479

Water, simple process for purifying 191

———, its efficacy in influenza 228

——— in scalds and ophthalmia 239

——— pepper, botanical description of 546

Waterhouse, Dr. on the vaccine virus 283

———, his memoir of Dr. Jenner, noticed 408

Westmeath's, Earl of, letter to Dr. Jenner 256

Westminster hospital, medical instruction at 286

Weston's, Mr. case of tinea capitis 305

Whateley's, Mr. cases of polypi of the nose 179

White lead, method of making 383

White's, Dr. case of diseased brain 247

Whitlam, Mr. on the use of conium maculatum 125

———, on galvanism in epilepsy 527

Whortle-berries, description and use of 173

Wilkinson's, Mr. answer to Mr. Cuming 46

———, on cuprum ammoniacum 47

Willan, Dr. on cutaneous diseases, reviewed 362

———, on inoculation 311

———, on prurigo 525

Willow-herb, botanical description of 171

Wilson's, Mr. lectures announced 288

Winterbottom, Dr. on inoculation 22

Wood's, Dr. case of tumour of the brain 80

Woodville, Dr. on the death of 83

Worms, good remedy for 545

Writing, position to be used in 13

Wurmser, Gen. on the skull of 332

Yeast, substitute for 546

Yelloly's, Dr. lectures announced 383

Yellow fever, resuscitation in a case of supposed death from 7

——— of America, observations on the 79

——— cured by sweating in the steam of sugar 185

———, preventive against the 205

——— at Catskill, origin and progress of the 312

———, on the contagiousness or non-contagiousness of the 385

———, prize questions on the 381

———, Dr. Brown's letter to Dr. Dancer on 389

———, Dr. Lafuente's method of curing the 531

Young's, Mr. treatise on cancer, reviewed 465

———, description of the canulated catheter 490

——— lectures on cancer announced 576

Zoroaster, account of a pretended manuscript of 91

### REFERENCES TO THE PLATES.

Mr. Ring's Case of confluent Chicken-pox – – – – – – 141
Mr. Young's Canulated Catheter }
Mr. Hall's Screw Tourniquet } – – – – – – – – 490

